# The Anatomy and Pathology of the Teeth

**Published:** 1895-06

**Authors:** 


### Bibliographical.

THE ANATOMY AND PATHOLOGY OF THE TEETH. By C. F. W. Bodecker, D. D. S., M. D. S.—With three hundred and twenty-five illustrations. Publishers: The S. S. White Dental Manufacturing Co.

Among the valuable contributions to the literature of the science of dentistry, this work of Dr. Bodecker will occupy a most prominent place, and be accepted by the dental profession as a clear and comprehensive teacher on dental pathology.

It abounds in original investigations by the author on subjects of the greatest interest to the dentist, together with those of other dental scientists who have, like Dr. Bodecker, been the pupils of Dr. Carl Heitzmann.

Dr. Bodecker in this work adopts the views of Dr. Heitzmann concerning the cell-theory, and believes that at no distant day the novel views of his preceptor and teacher, which are entirely antagonistic to that theory, will gain a final victory.

This valuable work of Dr. Bodecker begins with a description of the superior and inferior maxillary bones, the teeth, their articulation and general anatomy. The general histology of the teeth follows, and includes a number of chapters in which this subject is treated in a manner which establishes the high intellectual acquirements of the author and his untiring zeal and devotion to microscopical investigation and study. This portion of the work also abounds in beautiful illustrations of the minute structure and development of dentine, enamel and cementum. Faulty tooth-structures also receive careful attention, followed by the histology and structure of the dental pulp, the pericementum, absorption of temporary teeth, the physiology of the hard dental tissues.

The pathology of the teeth is then referred to, beginning with the general diagnosis of the diseases of the teeth, irritation and new formation of dental tissue, secondary dentine, mechanical abrasion, erosion, reaction of the dentine upon fillings, hyperostosis of roots of teeth, inflammation of gums and of dentine, pulpitis in its clinical aspects and morbid anatomy, degenerations and atrophies. The chapter on pericementitis is an exceedingly interesting and instructive treatise, referring to its clinical aspects and morbid anatomy. The chapter on caries of the teeth is a very excellent treatise of this subject and worthy of careful perusal. The effects of arsenic upon the pulps of the teeth follows, together with necrosis of the jaw-bones, empyemia of the antrum, clinical and anatomical features of tumors, especially tumors of the teeth and jaws, and cysts in the oral cavity, the contents of this work ending with malformation and mal-positions of the teeth.

While some may adhere to the cell doct.ine or theory, and others may regard some of the subdivisions of pericementitis, for example, as strictly characterizing advanced stages of this affection which should be classified under alveolar abscess, etc., yet all must admit that the subject matters embraced in this voluminous treatise have been treated in a manner worthy of universal admiration. This work comprises 676 pages, and the many excellent illustrations add very much to its great value and comprehension.

